# Genome Sequence of *Weissella ceti* NC36, an Emerging Pathogen of Farmed Rainbow Trout in the United States

**DOI:** 10.1128/genomeA.00187-12

**Published:** 2013-02-14

**Authors:** Jason T. Ladner, Timothy J. Welch, Chris A. Whitehouse, Gustavo F. Palacios

**Affiliations:** a Center for Genomic Sciences, U.S. Army Medical Research Institute of Infectious Diseases, Fort Detrick, Maryland, USA; b National Center for Cool and Cold Water Aquaculture, Agricultural Research Service/U.S. Department of Agriculture, Kearneysville, West Virginia, USA

## Abstract

Novel *Weissella* sp. bacteria have recently been reported to be associated with disease outbreaks in cultured rainbow trout (*Oncorhynchus mykiss*) at commercial farms in China, Brazil, and the United States. Here we present the first genome sequence of this novel *Weissella* species, isolated from the southeastern United States.

## GENOME ANNOUNCEMENT

The genus *Weissella* consists of Gram-positive, catalase-negative, non-spore-forming, nonmotile bacteria with irregular or coccoid heterofermentative rod morphologies (1). *Weissella* species are common in diverse nutrient-rich environments, including fermented foods, soil, and the intestines of many animals, including humans (2). *Weissella confusa* strains have infrequently been reported to cause infections in both humans (3–5) and nonhuman primates (6); however, members of the genus are not typically associated with disease. Novel *Weissella* sp. bacteria have recently been associated with disease outbreaks in rainbow trout (*Oncorhynchus mykiss*) in China (7), Brazil (8), and the United States (T. J. Welch and C. M. Good, submitted for publication). Each of these outbreaks occurred at commercial rainbow trout farms and caused high levels of morbidity and mortality. The origin of the bacteria associated with these outbreaks is unknown, but 16S rRNA sequences from the Brazilian, Chinese, and U.S. isolates are >99% identical, suggesting a high level of genetic similarity among strains (Welch and Good, submitted). The trout isolates also show >99% 16S sequence similarity to *W. ceti* sp. nov., which was recently isolated from beaked whales (9), and therefore, the whale and fish isolates may constitute a single species. The occurrence of this pathogen on three continents over a relatively short period (5 years) suggests that weissellosis is a rapidly emerging disease of farmed rainbow trout. Comparison of the genome sequences of the U.S., Brazilian, and Chinese strains will be necessary to our understanding of the evolutionary relationship among the strains and may additionally provide insight into the recent emergence of this pathogen. As a basis for these comparisons, and to identify putative virulence genes, we sequenced the genome of *Weissella ceti* NC36, a representative strain from the U.S. outbreak.

Genomic DNA was purified by using the MasterPure Gram-positive DNA purification kit (Epicenter) according to the supplied protocol. The genome was assembled by using a combination of sequences from Illumina (MiSeq, paired-end 150-bp reads; 583× coverage) and Pacific Biosciences (PacBio RS 10-kb library of continuous long reads [CLR]; 198× coverage). An initial assembly was conducted using ABySS (10) with only the Illumina sequences, resulting in 23 high-quality contigs of ≥500 bp. The PacBio CLR sequences were then leveraged to join these contigs together using AHA (Pacific Biosciences). Finally, small assembly errors were corrected through an iterative process of mapping the Illumina reads onto the final contigs and then creating a new consensus using Bowtie2, Samtools, and custom scripts (11, 12). The final assembly consisted of seven contigs (*N*_50_, 385,673 bp; maximum length, 518,056 bp), and the genome was estimated to be ~1.35 Mb, with a G+C content of 40.8%. Automated annotation by NCBI’s Prokaryotic Genomes Automatic Annotation Pipeline (PGAAP) revealed 16 rRNA genes, 68 tRNA genes, and 1,264 protein-coding sequences (CDS).

Results of comparative analysis highlighted several putative virulence factors, which do not have homologs encoded in any of the other sequenced *Weissella* genomes. These include five collagen adhesins (WCNC_00912, WCNC_00917, WCNC_00922, WCNC_05547, and WCNC_06207), a platelet-associated adhesin (WCNC_01820), and a mucus-binding protein (WCNC_01840).

### Nucleotide sequence accession numbers.

This Whole Genome Shotgun project has been deposited at DDBJ/EMBL/GenBank under the accession number ANCA00000000. The version described in this paper is the first version, ANCA01000000.

## References

[1] Collins MD Samelis J Metaxopoulos J Wallbanks S 1993 Taxonomic studies on some leuconostoc-like organisms from fermented sausages: description of a new genus Weissella for the Leuconostoc paramesenteroides group of species. J. Appl. Bacteriol. 75:595–603 8294308 10.1111/j.1365-2672.1993.tb01600.x

[2] Björkroth KJ Schillinger U Geisen R Weiss N Hoste B Holzapfel WH Korkeala HJ Vandamme P 2002 Taxonomic study of Weissella confusa and description of Weissella cibaria sp. nov., detected in food and clinical samples. Int. J. Syst. Evol. Microbiol. 52:141–148 11837296 10.1099/00207713-52-1-141

[3] Olano A Chua J Schroeder S Minari A La Salvia M Hall G 2001 Weissella confusa (basonym: Lactobacillus confusus) bacteremia: a case report. J. Clin. Microbiol. 39:1604–1607 11283096 10.1128/JCM.39.4.1604-1607.2001 PMC87979

[4] Flaherty JD Levett PN Dewhirst FE Troe TE Warren JR Johnson S 2003 Fatal case of endocarditis due to Weissella confusa. J. Clin. Microbiol. 41:2237–2239 12734290 10.1128/JCM.41.5.2237-2239.2003 PMC154740

[5] Lee MR Huang YT Liao CH Lai CC Lee PI Hsueh PR 2011 Bacteraemia caused by Weissella confusa at a university hospital in Taiwan, 1997–2007. Clin. Microbiol. Infect. 17:1226–1231 21040157 10.1111/j.1469-0691.2010.03388.x

[6] Vela AI Porrero C Goyache J Nieto A Sánchez B Briones V Moreno MA Domínguez L Fernández-Garayzábal JF 2003 Weissella confusa infection in primate (Cercopithecus mona). Emerg. Infect. Dis. 9:1307–1309 14626220 10.3201/eid0910.020667 PMC3033088

[7] Liu JY Li AH Ji C Yang WM 2009 First description of a novel Weissella species as an opportunistic pathogen for rainbow trout Oncorhynchus mykiss (Walbaum) in China. Vet. Microbiol. 136:314–320 19321272 10.1016/j.vetmic.2008.11.027

[8] Figueiredo HC Costa FA Leal CA Carvalho-Castro GA Leite RC 2012 Weissella sp. outbreaks in commercial rainbow trout (Oncorhynchus mykiss) farms in Brazil. Vet. Microbiol. 156:359–366 22137197 10.1016/j.vetmic.2011.11.008

[9] Vela AI Fernandez A de Quiros YB Herraez P Dominguez L Fernandez-Garayzabal JF 2011 Weissella ceti sp. nov., isolated from beaked whales (Mesoplodon bidens). Int. J. Syst. Evol. Microbiol. 61:2758–2762 21216921 10.1099/ijs.0.028522-0

[10] Simpson JT Wong K Jackman SD Schein JE Jones SJ Birol I 2009 ABySS: a parallel assembler for short read sequence data. Genome Res. 19:1117–1123 19251739 10.1101/gr.089532.108 PMC2694472

[11] Langmead B Salzberg SL 2012 Fast gapped-read alignment with bowtie 2. Nat. Methods 9:357–359 22388286 10.1038/nmeth.1923 PMC3322381

[12] Li H Handsaker B Wysoker A Fennell T Ruan J Homer N Marth G Abecasis G Durbin R 1000 Genome Project Data Processing Subgroup 2009 The sequence alignment/map format and SAMtools. Bioinformatics 25:32078–2079 10.1093/bioinformatics/btp352 PMC2723002 19505943

